# Nano-Biosensors for Detection and Monitoring (Volume 1)

**DOI:** 10.3390/bios13110966

**Published:** 2023-11-01

**Authors:** Krishna Kant

**Affiliations:** 1 Biomedical Research Center (CINBIO), University of Vigo, 36310 Vigo, Spain; krishna.kant@uvigo.gal; 2 Centre for Interdisciplinary Research and Innovation (CIDRI), University of Petroleum and Energy Studies, Dehradun 248007, India

Nano-biosensing technology is a continuously evolving and expanding field with applications concerning biological substances and sensing platforms, which include the detection of chemical, biological, and environmental elements and welfare. However, sensing biological analytes is of significance in the diagnostic industry. Biosensing analyses are required in areas like pharmaceutical, food industry, and environmental applications. The development of capable biosensors is important, and they can rapidly recognize biological interactions at small scales with maximum precision. Nanomaterials, along with biosensing technology, enhanced the capabilities of biosensors with respect to their high surface area-to-volume ratios and functional capabilities. These types of sensors are usually used as bioreceptors at bio–nano interfaces. Numerous kinds of nanostructures involving nanoparticles, nanotubes, nanowires, and nanocomposites are effectively used to enhance the operation and productivity of sensors. Concurrently, the use of nanostructures and sensing technologies led to the development of biosensors with high specificity and compatibility. Biosensing technology provides the benefits of nanoscience and offers the potential to develop monitoring sensors for the early-stage diagnosis of diseases in an inexpensive manner.

This Special Issue is devoted to and collected advances in a variety of topics: from recognition to engineering and from integration methods to novel sensors. Articles reporting on the newest advances in multiplexed detection report on electrochemical, optical, and magnetic biosensing platforms, in addition to others. An interesting article by Jeong Hee Kim et al. (contribution 1) presented a novel approach for the non-perturbative identification of kidney tissue using Raman spectroscopy and artificial intelligence. The integration of sophisticated and sensitive Raman techniques with AI-based approaches is leading to the development of a model system for accurate detection, and these techniques are used for point-of-care purposes as well. In their approach, they employ Raman spectroscopy and machine learning algorithms, which present the capability for the recognition of amyloids in pathologic lesions. Sara Knežević et.al. (contribution 2) presented a multi-walled carbon nanotube-based sensing approach for the recognition of uric acid. They developed an amperometric, non-enzymatic sensor for the sensitive quantification of uric acid. The developed sensing device presents high reproducibility and a limit of detection up to 64.28 nM. The sensing device is also tested with real-life samples and shows high sensitivity and stability, which makes it a potential candidate for further medical research and clinical application. This Special Issue collected innovative approaches for detection and commercial applications with respect to clinical and environmental samples. The development approaches and recent advancements have also been discussed for nanobiosensors and their detection and monitoring approaches. These arrangements are very crucial for clinical diagnostic applications, and they are required for sample arrangement, sensing, and data administration. Minimizing technology usage via the minimum mandatory sample size is appropriate for the analysis of samples as it would not require skilled users.

